# Trifluoromethylation‐Induced Lactonization: A Novel Transformation of Unsaturated Carboxylic Acids

**DOI:** 10.1002/open.70273

**Published:** 2026-08-02

**Authors:** Attila M. Remete

**Affiliations:** ^1^ Institute of Pharmaceutical Chemistry University of Szeged Szeged Hungary

**Keywords:** lactonization, photoredox catalysis, radical reactions, trifluoromethylation

## Abstract

Fluorinated drugs are highly important, and one of their common structural motifs is the trifluoromethyl group. As a result, there is an increasing demand for new or improved synthetic pathways toward trifluoromethylated compounds. One such route, developed less than one and a half decades ago, is trifluoromethylation‐induced lactonization of unsaturated carboxylic acids. Initial successes (including the only known enantioselective protocol for this reaction) were achieved using Togni's reagent II in the presence of a Cu(I) catalyst (mechanism: radical trifluoromethylation, then Cu‐assisted lactonization), then the focus shifted to photocatalytic methods (mechanism: radical trifluoromethylation, single‐electron oxidation, then lactonization). Later, electrocatalytic methods were developed too (mechanism: radical trifluoromethylation, single‐electron oxidation, then lactonization). Recently, the first CF_3_^+^ transfer‐induced lactonization was finally realized as well. The purpose of this review is to provide a comprehensive overview of the current state of this field of research. First, the importance of trifluoromethylation, CF_3_‐containing drugs, and lactonizations is explained briefly. Then, possible reaction mechanisms of trifluoromethylation‐induced lactonizations are discussed. Finally, I will present known trifluoromethylative lactonizations, grouped according to their mechanisms and the type of chemistry involved.

## Graphical abstract

Fluorinated drugs often contain trifluoromethyl groups. The increasing importance of such drugs has resulted in more and more attention toward new or improved trifluoromethylation reactions. This review discusses possible reaction mechanisms and existing methods for one such process, trifluoromethylation‐induced lactonization, a distant relative of the versatile and widely utilized iodolactonization.

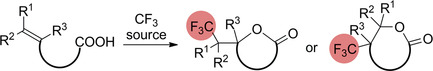

## 1 Introduction

In the 1950s, fluorine‐containing drugs were a rarity [1]. Nowadays, such compounds are surprisingly common (25%–30% of recently approved drugs contain fluorine) [2, 3]. The reasons behind the increasing prevalence of fluorine in drugs are complex [3, 4, 5, 6]. In short, the fluorine atom and the C—F bond have a number of unique features, which often enhance pharmacologically relevant properties of organofluorine compounds [3, 4, 5, 6].

**FIGURE 1:**
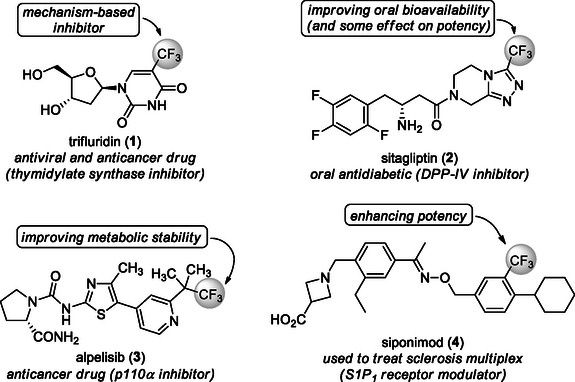
Examples of trifluoromethylation‐related benefits in drugs.

One of the common fluorinated motifs of drug molecules is the trifluoromethyl group [2, 3]. From a medicinal chemistry perspective, the most important properties of the CF_3_ group are its high lipophilicity (importantly, lipophilicity is a key pharmaceutical parameter which strongly influences membrane penetration and oral bioavailability) [6, 7], surprising bulkiness (based on its Charton steric parameter and revised Taft steric parameter, it is bulkier than an *i*Pr group, and almost as bulky as an *i*Bu group) [6], and excellent chemical stability [8, 9]. Figure 1 shows some examples where the introduction of a CF_3_ group proved to be advantageous [3].

**FIGURE 2:**
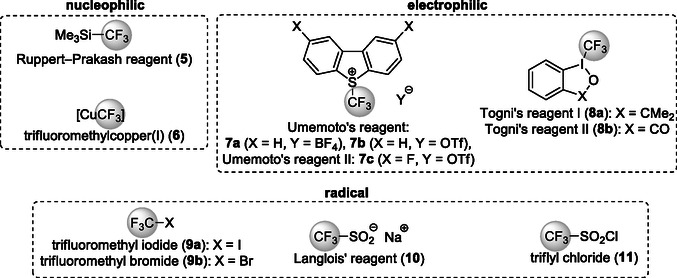
The most important current trifluoromethylating reagents.

Electrochemical fluorination [10, 11] and Lewis acid‐assisted halogen→fluorine exchange [12], the earliest methods to produce trifluoromethylated molecules [13], suffering from a narrow substrate scope and low functional group tolerance. Therefore, as the importance of trifluoromethylated compounds in medicinal chemistry grew, more and more efforts were directed to develop new, improved synthetic pathways toward such molecules. Nowadays, numerous nucleophilic [14, 15, 16, 17], electrophilic [8, 16, 17, 18], radical [7, 16, 17, 19], oxidative [16, 17, 20], or transition metal‐catalyzed [16, 17, 20] trifluoromethylation methods are known. The most common trifluoromethylating reagents are depicted in Figure 2.

**SCHEME 1:**
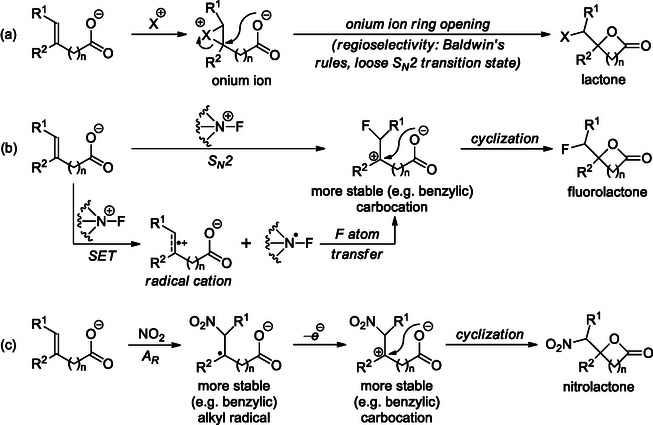
Mechanisms of various lactonizations of olefincarboxylic acids. (a) Most common mechanism (X = Cl, Br, I, SR, SeR). (b) Mechanisms of Selectfluor‐mediated fluorolactonization (without an aryl iodide or transition metal catalyst). (c) Mechanism of Fe(NO_3_)_3_‐mediated nitrolactonization.

Iodolactonization is a long‐known, highly versatile olefin difunctionalization reaction, which is widely used in organic synthesis. Under the usual conditions (base and protic solvent), the first step is attack of an electrophilic iodine species on the double bond. Then, the resulting iodonium ion reacts with the pendant carboxylate group, resulting in iodonium ion ring opening (with a regioselectivity which is influenced by Baldwin's rules and the “loose S_*N*_2” nature of the transition state) and closure of the lactone ring [21, 22]. The great success of iodolactonization reactions resulted in the development of a number of analogous processes. Chloro‐ and bromolactonizations [21], sulfenyllactonizations [23, 24, 25], and selenolactonizations [23, 26, 27] seem to follow the same mechanism as iodolactonization. In the absence of catalysts (aryl iodides or transition metals), Selectfluor‐mediated fluorolactonizations [28, 29] follow a slightly different pathway: first, the alkene is transformed into a β‐fluorinated carbocation (either by single electron transfer and subsequent F atom transfer, or by ionic “F^+^” transfer) [28], then the carbocation undergoes lactone ring closure. For Fe(NO_3_)_3_‐mediated nitrolactonizations, a third pathway was suggested: addition of NO_2_ (a free radical) to the alkene generates a β‐nitro radical, which is oxidized into a β‐nitro carbocation, then ionic lactone ring closure yields the desired product [30]. The proposed mechanisms of the abovementioned lactonizations are summarized in Scheme 1.

As depicted in Scheme 1, lactonizations of alkenoic acids can be initiated by electrophiles or radicals. Taking into account the development of efficient electrophilic [8, 16, 17, 18] and radical [7, 16, 17, 19] trifluoromethylation reactions, the interest in new pathways toward trifluoromethylated compounds, and the popularity of the abovementioned lactonizations of alkenoic acids [21, 22, 23, 24, 25, 26, 27, 28, 29, 30], a logical question arises: is it possible to perform trifluoromethylation‐induced lactonization of olefincarboxylic acids? The question was answered in 2012: yes, it is possible [31]. Since then, almost one and a half decade has passed, and now a handful of protocols are known [32, 33, 34, 35, 36, 37, 38, 39, 40, 41, 42, 43, 44, 45, 46, 47, 48, 49, 50]. The purpose of this Review is to provide a comprehensive overview of the current state of this field of research. First, based on mechanistic investigations of trifluoromethylation‐induced lactonizations [33, 34, 35, 37, 40, 41, 44, 45, 47, 48, 50], possible reaction mechanisms will be discussed. Then, known methods for trifluoromethylation‐induced lactonizations will be described, grouped according to their possible mechanisms and the type of chemistry involved [31, 32, 33, 34, 35, 36, 37, 38, 39, 40, 41, 42, 43, 44, 45, 46, 47, 48, 49, 50].

## 2 Mechanistic Considerations

**SCHEME 2:**
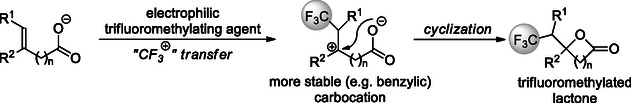
Mechanism of “electrophilic” trifluoromethylation‐induced lactonizations of olefincarboxylic acids.

As depicted in Scheme 1, most lactonizations of alkenoic acids start with the attack of an electrophile on the alkene, resulting in an intermediate that is either an onium ion with a strained three‐membered ring or a carbocation (depending on the nature of the electrophile). Electrophilic trifluoromethylating agents are known (see Figure 2), so a “CF_3_^+^”‐induced lactonization is theoretically possible (note: CF_3_^+^ transfer is more likely than involvement of free CF_3_^+^ cations), and it would follow the same pathway as S_*N*_2‐initiated fluorolactonizations (see Scheme 1b), because the CF_3_ cation cannot support an onium ion intermediate. However, to date, there is only one known reaction [50] which definitely follows this pathway (Scheme 2).

**SCHEME 3:**
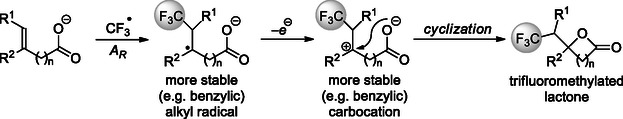
Mechanism of the majority of CF_3_ radical‐induced lactonizations of olefincarboxylic acids.

The nitrolactonization depicted in Scheme 1c clearly indicates that CF_3_ radical‐induced lactonizations should be possible too. Luckily, the CF_3_ radical is formed relatively easily via a variety of pathways. Notably, even reactions involving dedicated electrophilic trifluoromethylating reagents often follow a radical pathway (single‐electron reduction of reagents **7**‐**8** efficiently provides CF_3_ radicals) [8, 19]. With these in mind, it is no longer surprising that the majority of mechanistic investigations of trifluoromethylation‐induced lactonizations found a radical‐induced mechanism [33, 34, 35, 36, 37, 40, 41, 44, 45, 47, 48]. In fact, there are good reasons to suspect that many of those trifluoromethylation‐induced lactonizations, whose mechanism was not investigated in detail [38, 39, 42, 43, 46, 49], are CF_3_ radical‐induced too (see Section 3.2 below for more details). The simplest pathway for CF_3_ radical‐induced lactonization, which involves unassisted C—O bond formation [37, 40, 41, 44, 45, 47, 48], is depicted in Scheme 3.

**SCHEME 4:**
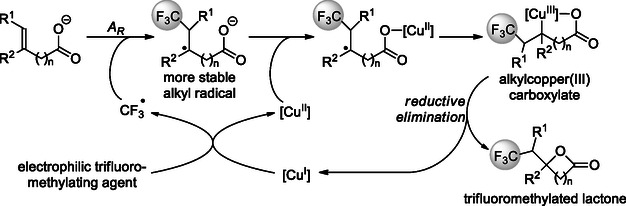
Mechanism of a CF_3_ radical‐induced lactonization with copper‐catalyzed C—O bond formation.

There are a number of aryltrifluoromethylations and alkenyltrifluoromethylations which are CF_3_ radical‐induced, but the trifluoromethylated alkyl radical intermediate is captured by an aryl‐ or alkynylcopper(II) species, forming a Cu(III) intermediate. Importantly, in the presence of chiral ligands, these processes were capable of enantioselective transformations [51, 52]. Because single electron transfer between electrophilic trifluoromethylating regents and Cu(I) compounds provides both CF_3_ radicals and Cu(II) compounds, subjecting an alkenoic acid to such a system may trigger a CF_3_ radical‐induced lactonization with Cu‐catalyzed C—O bond formation (Scheme 4). The first reported method for trifluoromethylation‐induced lactonization [31] followed this mechanism [33, 34], enabling the development of an enantioselective variant of that protocol [33]. Up to date, the latter process is the only known asymmetric method for trifluoromethylation‐induced lactonization.

## 3 Review of Literature Protocols

### 3.1 CF_3_ Radical‐Induced Lactonizations in the Presence of Copper Species

Amongst trifluoromethylation‐induced lactonizations, these protocols have special importance: the first reported method [31] as well as the only enantioselective method [33] belong to this group. That justifies discussing them first.

**SCHEME 5:**
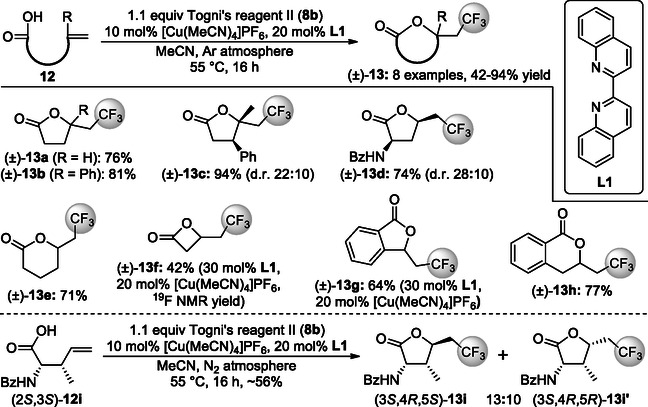
Racemic trifluoromethylation‐induced lactonization of terminal alkenoic acids using Togni's reagent II, a Cu(I) salt, and an achiral ligand. In the cases of diastereomeric mixtures, only structures of the major diastereomers are shown. Isolated yields are provided, unless it is noted otherwise.

In 2012, Zhu and Buchwald reported intramolecular oxytrifluoromethylation of alkenes using Togni's reagent II in the presence of [Cu(MeCN)_4_]PF_6_ catalyst and 2,2′‐biquinoline ligand. Most substrates were carboxylic acids with a terminal olefin motif (Scheme 5, top and middle), but the reaction also worked on unsaturated alcohols and 2‐allylphenol. Low yields were obtained with 1,2‐disubstituted alkene substrates [31]. In 2024, Wendlandt and coworkers used the same protocol for a single trifluoromethylation‐induced lactonization (Scheme 5, bottom) [32]. Zhu and Buchwald reported that the addition of the radical scavenger 2,2,6,6‐tetramethylpiperidine‐1‐oxyl (TEMPO) completely inhibited the process and concluded that radicals are involved in the reaction [31] (although it was pointed out by Togni and coworkers in 2015 that Togni's reagent II can trifluoromethylate TEMPO even in the absence of additives) [18]. Later, thorough mechanistic studies of a slightly modified version of the protocol proved that the mechanism involves copper‐catalyzed C—O bond formation, and the reaction follows the pathway depicted on Scheme 4 [33, 34].

**SCHEME 6:**
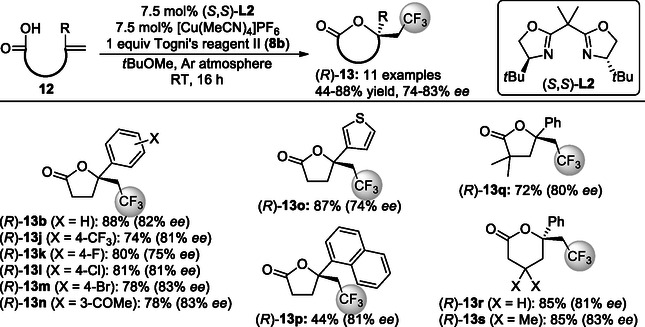
Asymmetric trifluoromethylation‐induced lactonization of terminal alkenoic acids using Togni's reagent II, a Cu(I) salt, and a chiral ligand. Isolated yields are provided.

**SCHEME 7:**
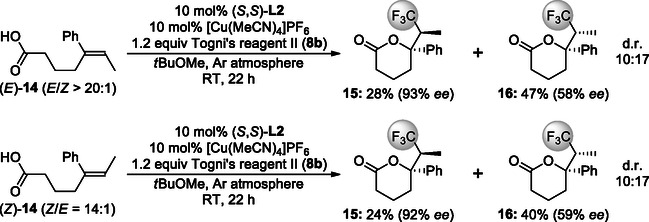
Asymmetric trifluoromethylation‐induced lactonization of trisubstituted alkenes. ^19^F NMR yields are provided.

In 2013, Zhu and Buchwald reported an enantioselective version of the above protocol. They achieved this by replacing achiral 2,2′‐biquinoline ligand **L1** with chiral bisoxazoline ligand (*S*,*S*)‐**L2** and changing the solvent from MeCN to *t*BuOMe. Most substrates were carboxylic acids with an arylated terminal olefin motif (Scheme 6), but substrates (*E*)‐**14** and (*Z*)‐**14** were also transformed successfully (Scheme 7). The latter reactions proved that formation of the C—O bond is stereoselective (mainly influenced by the chiral ligand, but somewhat affected by the configuration of the trifluoromethylated carbon as well), but formation of the C—CF_3_ bond is not. Experiments with a radical clock and with diallylmalonic acid diethyl ester supported the intermediacy of CF_3_ radicals [33]. After further studies, the authors have concluded that the process follows the pathway depicted in Scheme 4 (C—CF_3_ bond formation: A_R_, C—O bond formation: copper‐catalyzed) [34].

After carefully reviewing the literature, two more cases were found where CF_3_ radical‐induced lactonization happened in the presence of a copper species. In these cases, the involvement of copper species in the C—O bond formation was not investigated (therefore it is neither proven nor disproven), but for the sake of simplicity, they are included in this section [35, 36].

**SCHEME 8:**
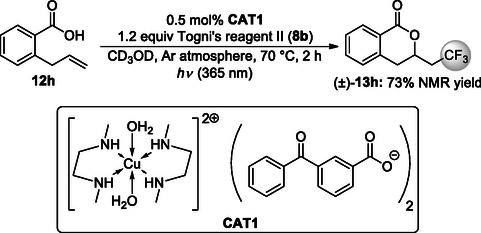
Trifluoromethylation‐induced lactonization using Togni's reagent II and a photoreducible Cu(II)‐complex.

In 2015, Vincent and coworkers reported photocatalyzed trifluoromethylation of alkenes in methanol (which served both as solvent and sacrificial electron source) using Togni's reagent II, a photoreducible Cu(II) complex, and irradiation with sunlight (or 365 nm UV light). According to mechanistic investigations, at first, irradiation excites the benzophenone carboxylate, which results in photoinduced electron transfer from the solvent to the Cu(II) center. Afterward, the formed Cu(I) species reduces Togni's reagent II (this is facilitated by coordination of an oxygen atom of Togni's reagent II to a copper ion), producing CF_3_ radicals, which perform A_R_ on the alkene. The formed trifluoromethylated alkyl radical is usually stabilized by hydrogen abstraction (proposed to happen via an alkylcopper(III) complex), but in the presence of a pendant nucleophile, the alkyl radical can undergo cyclization instead. A single trifluoromethylation‐induced lactonization was reported (Scheme 8) [35].

**SCHEME 9:**
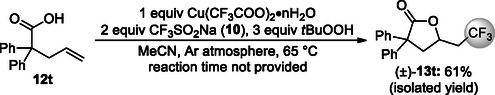
Trifluoromethylation‐induced lactonization using CF_3_SO_2_Na, *t*BuOOH, and a stoichiometric amount of Cu(II) salt.

Oxidation of CF_3_SO_2_Na is a well‐established pathway toward CF_3_ radicals [7]. In 2017, Zhang and coworkers reported intramolecular aminotrifluoromethylation of alkenes using CF_3_SO_2_Na and *t*BuOOH oxidant in the presence of a stoichiometric amount of Cu(II) trifluoroacetate hydrate (reducing the amount of Cu(II) salt greatly decreased the yield). Addition of radical scavengers like TEMPO or 1,1‐diphenylethylene suppressed product formation (in the presence of the latter, 1,1‐diphenyl‐2‐trifluoromethylethylene was formed instead), confirming the involvement of CF_3_ radicals in the process, but further mechanistic details (e.g., the exact mechanism of ring closure) are unknown. In addition to numerous aminotrifluoromethylations, a single trifluoromethylation‐induced lactonization (Scheme 9) was also reported [36].

### 3.2 CF_3_ Radical‐Induced Lactonizations in the Absence of Copper Species

According to mechanistic investigations, trifluoromethylation‐induced lactonizations which take place in the absence of a copper catalyst almost always follow the radical‐induced mechanism depicted on Scheme 3 [37, 40, 41, 44, 47, 48]. There is one partial exception (it follows a slightly different but still CF_3_ radical‐induced mechanism, see Schemes 20 and 21 for further details) [45], and one complete exception (it follows a fully ionic mechanism, see Scheme 26 for further details) [50]. Furthermore, many trifluoromethylative lactonizations, whose mechanism was not investigated in detail [38, 39, 42, 43, 46, 49], utilize conditions which readily generate CF_3_ radicals, strongly suggesting that these reactions also follow the radical‐induced mechanism depicted on Scheme 3. Therefore, the protocols included in this section represent the most common method for trifluoromethylation‐induced lactonization.

The protocols are grouped based on how they generate the necessary CF_3_ radical. There is only one method that generates CF_3_ radicals in a “traditional” way (using a stoichiometric amount of a chemical redox agent) [37], which will be discussed first. Afterward, the seven methods which utilize photoredox catalysis [38, 40, 41, 42, 43, 44, 45] will be discussed. The remaining four methods are electrochemical [46, 47, 48, 49]; these will be discussed last.

**SCHEME 10:**
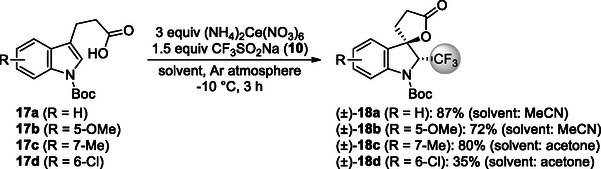
Trifluoromethylation‐induced lactonization 3‐(indol‐3‐yl)propanoic acid derivatives using CF_3_SO_2_Na and (NH_4_)_2_Ce(NO_3_)_6_. Isolated yields are provided.

In 2017, Vincent and coworkers reported radical‐mediated dearomatization of indoles with a pendant nucleophilic group using fluorinated alkanesulfinates (mainly CF_3_SO_2_Na) as radical sources and (NH_4_)_2_Ce(NO_3_)_6_ as an oxidant. Apart from 3‐(indol‐3‐yl)propane‐1‐ols and *N*‐tosylated 3‐(indol‐3‐yl)propane‐1‐amines, four 3‐(indol‐3‐yl)propanoic acid derivatives were also transformed successfully (Scheme 10) [37]. Because oxidation of CF_3_SO_2_Na is a well‐established pathway toward CF_3_ radicals [7], and no fluorinated compound is formed if radical scavenger TEMPO is added to the reaction mixture, the most plausible mechanism is the one depicted on Scheme 3 (although the oxidizable nature of the indole substrates allows another mechanism too) [37]. Importantly, according to ^1^H NMR of the crude mixture after aqueous workup, all products were formed as single diastereomers, which was supported by X‐ray analysis of the crystals of one product [37]. However, it should be noted that according to Gao, Zhu, and coworkers, trifluoromethylation‐induced cyclizations of analogous compounds produce diastereomeric mixtures whose minor component is difficult to detect with ^1^H NMR, necessitating the use of SFC‐MS to determine d.r. [49].

**SCHEME 11:**
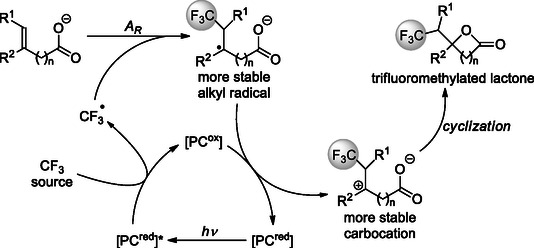
Most common mechanism of CF_3_ radical‐induced lactonization with photocatalysis. [PC^red^] is the reduced photocatalyst (in the ground state), [PC^red^]* is the reduced photocatalyst (in the excited state), [PC^ox^] is the oxidized photocatalyst. Note that the process is redox‐neutral.

As mentioned at the beginning of this section, the majority of copper‐free CF_3_ radical‐induced lactonizations (seven methods out of twelve) utilize photocatalysis. To understand the reasons behind this popularity, we have to take a look at the mechanism. All seven photocatalytic methods use a photocatalyst in the reduced state and a trifluoromethylating agent which that can release CF_3_ radicals upon single‐electron reduction [38, 40, 41, 42, 43, 44, 45]. According to mechanistic investigations [40, 41, 44, 45], upon excitation, the reduced photocatalyst transfers an electron to the CF_3_ source, resulting in a CF_3_ radical and the oxidized photocatalyst. This method for producing CF_3_ radicals can be integrated perfectly into the mechanism depicted on Scheme 3, because reaction of the oxidized photocatalyst with the trifluoromethylated alkyl radical intermediate (formed by A_R_ of the CF_3_ radical to the substrate) not only provides the trifluoromethylated carbocation (which then undergoes ring closure, finishing the reaction), but also regenerates the photocatalyst (Scheme 11) [40, 41, 44]. Of course, this requires a photocatalyst whose oxidized and reduced forms have appropriate redox potentials, so the whole reaction system has to be designed carefully; the choices of the photocatalyst and the trifluoromethylating reagent are especially important [39, 40]. Trifluoromethyl sources which are compatible with the mechanism depicted on Scheme 11 include all electrophilic trifluoromethylating agents (**7a‐c** and **8a‐b**, these are relatively expensive), trifluoromethyl halides (these are cheap, but gaseous at RT) and triflyl chloride (it is also cheap). Altogether, application of photoredox catalysis in trifluoromethylation‐induced lactonization greatly reduces waste formation (thanks to the redox‐neutral nature of the process, there is no need for stoichiometric amounts of oxidizing or reducing agents; note that “green chemistry” is an important goal nowadays) and enables the reaction to proceed under very mild conditions (no need for elevated temperature or harsh reagents, just some illumination).

**SCHEME 12:**
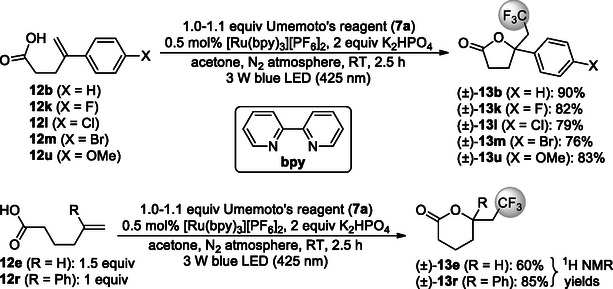
Trifluoromethylation‐induced lactonization of terminal alkenoic acids using [Ru^II^(bpy)_3_][PF_6_]_2_ photocatalyst and Umemoto's reagent. Isolated yields are provided unless it is noted otherwise.

**SCHEME 13:**
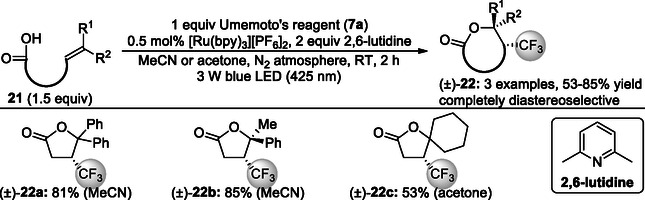
Trifluoromethylation‐induced lactonization of internal alkenoic acids (trisubstituted alkenes) using [Ru^II^(bpy)_3_][PF_6_]_2_ photocatalyst and Umemoto's reagent. Structure of **bpy** was depicted on Scheme 12. Isolated yields are provided.

**SCHEME 14:**
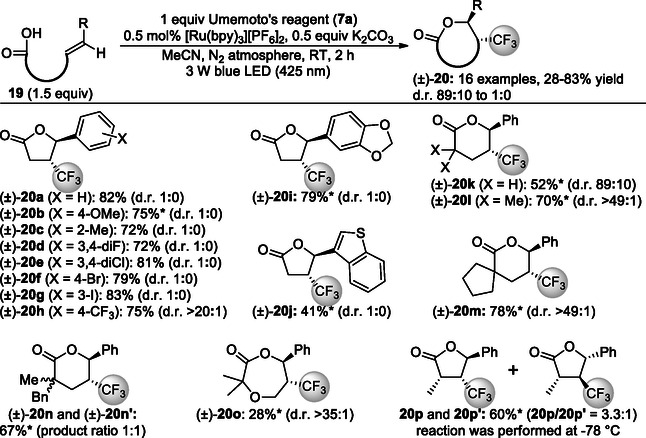
Trifluoromethylation‐induced lactonization of internal alkenoic acids (*trans* 1,2‐disubstituted alkenes) using [Ru^II^(bpy)_3_][PF_6_]_2_ photocatalyst and Umemoto's reagent. Structure of **bpy** was depicted on Scheme 12. Isolated yields are provided (yields marked with an asterisk were obtained in acetone as solvent). Diastereomeric ratios were determined by ^1^H and ^19^F NMR spectroscopies of crude reaction mixtures.

In 2014, Koike, Akita, and coworkers reported trifluoromethylative lactonization of both terminal and internal alkenoic acids utilizing Umemoto's reagent **7a** in the presence of 0.5 mol% [Ru^II^(bpy)_3_][PF_6_]_2_ photocatalyst and irradiation with 425 nm blue LEDs. 1,1‐disubstituted alkenes, *trans*‐1,2‐disubstituted alkenes, and trisubstituted alkenes required slightly different conditions (Schemes 12, 13–14). Transformation of internal alkenoic acids was highly diastereoselective: the bigger R group of the olefin and the CF_3_ group ended up *trans* to each other in the products. (In the cases of 4‐phenyl‐3‐butenoic acid substrates, DFT calculations revealed that product stereochemistry reflects the conformational preference of the trifluoromethylated carbenium ion intermediate.) On the basis of analogy with revious processes, the authors proposed the mechanism depicted in Scheme 11 [38]. The results were also included in an account which focused on photoredox‐catalyzed fluoromethylation of alkenes and alkynes [39].

**SCHEME 15:**
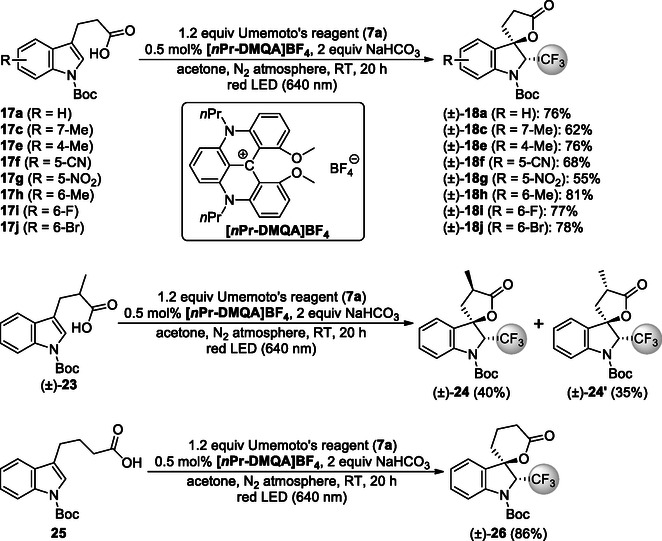
Trifluoromethylation‐induced lactonization of carboxylic acids with an indol‐3‐yl substituent using Umemoto's reagent and an organic photocatalyst. Isolated yields are provided.

Gianetti and coworkers reported cascade trifluoromethylation/dearomatization of indoles with a pendant nucleophilic group [3‐(indol‐3‐yl)propanoic acid derivatives, a 4‐(indol‐3‐yl)butanoic acid derivative, 3‐(indol‐3‐yl)propane‐1‐ols, and *N*‐tosylated 3‐(indol‐3‐yl)propane‐1‐amines] using Umemoto's reagent **7a** in the presence of organic photocatalyst **[*n*Pr‐DMQA]BF**_**4**_ and irradiation with 640 nm red LEDs (Scheme 15). The reaction only progressed upon illumination (note: irradiation with 467 nm blue LEDs was able to trigger the reaction even in the absence of **[*n*Pr‐DMQA]BF**_**4**_, while irradiation with 640 nm red LEDs led to transformation only in the presence of **[*n*Pr‐DMQA]BF**_**4**_), excluding a radical chain. The presence of radical scavenger TEMPO completely inhibited the reaction and led to formation of TEMPO‐CF_3_, proving the role of CF_3_ radicals in the reaction. Utilization of compounds where the pendant nucleophilic group was too close to the indole ring (or too far from it) resulted in only C2‐trifluoromethylation, suggesting a carbocation intermediate (which can stabilize through either cyclization or deprotonation). Thus, the authors proposed the mechanism depicted in Scheme 11. Importantly, the reaction proceeded in a completely diastereoselective manner, which was attributed to steric repulsion between the CF_3_ group and the 3‐substituent of the indole ring in the carbenium ion intermediate [40]. However, it should be noted that according to Gao, Zhu, and coworkers, trifluoromethylation‐induced cyclizations of analogous compounds produce diastereomeric mixtures whose minor component is difficult to detect with ^1^H NMR, necessitating the use of SFC‐MS to determine d.r. [49].

**SCHEME 16:**
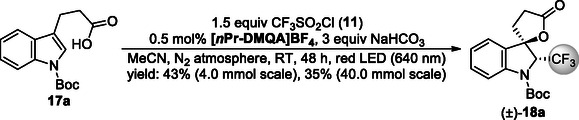
Trifluoromethylation‐induced lactonization of carboxylic acid **17a** using triflyl chloride and an organic photocatalyst. Isolated yields are provided. Structure of **[*n*Pr‐DMQA]BF**_**4**_ was depicted on Scheme 15.

Gianetti and coworkers also reported that changing the solvent to MeCN from acetone and increasing the amount of NaHCO_3_ enabled utilization of triflyl chloride as the CF_3_ source (Scheme 16). Although the obtained yields are lower, the cheapness of CF_3_SO_2_Cl still makes this modified protocol worthy of attention [40].

**SCHEME 17:**
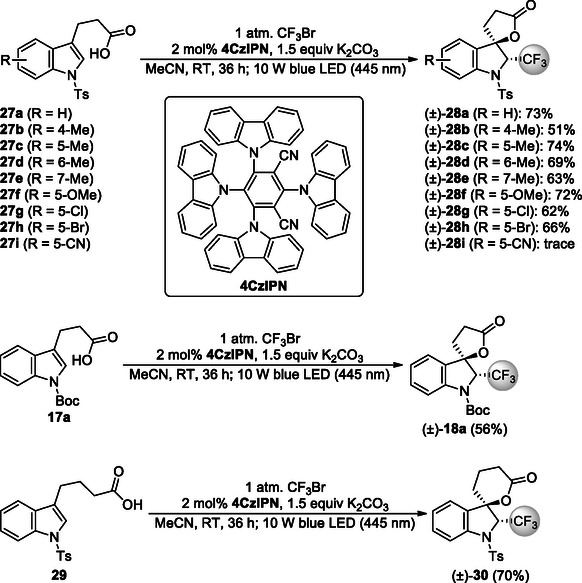
Trifluoromethylation‐induced lactonization of carboxylic acids with an indol‐3‐yl substituent using trifluoromethyl bromide and an organic photocatalyst. Isolated yields are provided.

Hu and coworkers reported cascade trifluoromethylation/dearomatization of indoles with a pendant nucleophilic group [3‐(indol‐3‐yl)propanoic acid derivatives, a 4‐(indol‐3‐yl)butanoic acid derivative, 3‐(indol‐3‐yl)propane‐1‐ols, and *N*‐tosylated 3‐(indol‐3‐yl)propane‐1‐amines] using trifluoromethyl bromide (**9b**) in the presence of organic photocatalyst **4CzIPN** and irradiation with 445 nm blue LEDs (Scheme 17) [41]. Note: CF_3_Br is a gas (b.p. −57.8 °C), which was used for fire suppression in significant quantities. However, because it has a detrimental effect on the ozone layer, its production was mostly phased out in 1994 as part of the Montreal Protocol [7, 53]. Still, CF_3_Br remained cheap and readily available in large quantities [54]. Control experiments proved that **4CzIPN**, a base, and irradiation are all necessary for successful transformation, and progress is halted if the blue LEDs are turned off. If radical scavengers (TEMPO or 2,6‐di‐tert‐butyl‐4‐methylphenol [BHT]) were added to the reaction mixtures, no product was formed, but HRMS and ^19^F NMR detected the CF_3_ adducts of the radical scavengers. Certain substrates underwent only C2‐trifluoromethylation [41]. Based on these observations and literature data, Hu and coworkers arrived at the same conclusion as Gianetti and coworkers [40]: the process follows the mechanism depicted on Scheme 11, and its complete diastereoselectivity is a consequence of the steric repulsion between the CF_3_ group and the 3‐substituent of the indole ring in the carbenium ion intermediate [41]. Note that Gao, Zhu, and coworkers reported that trifluoromethylation‐induced cyclizations of analogous compounds produce diastereomeric mixtures whose minor component is difficult to detect with ^1^H NMR, necessitating the use of SFC‐MS to determine d.r. [49].

**SCHEME 18:**
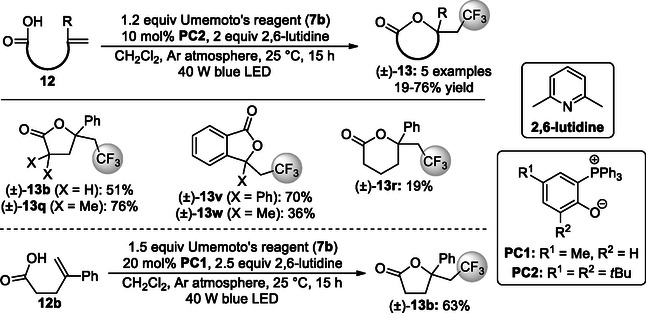
Trifluoromethylation‐induced lactonization of terminal alkenoic acids using Umemoto's reagent and an organic photocatalyst. Isolated yields are provided.

Toda et al. reported generation of HCl from CCl_3_CN using organic photocatalyst **PC1** and irradiation with blue LEDs. After control experiments proved that the reaction starts with single‐electron transfer from excited PC1* to CCl_3_CN (which generates a chloride ion and a •CCl_2_CN radical), the authors tried to combine their photoredox system with other reagents that release radicals upon single‐electron reduction. Using Umemoto's reagent **7b**, they reported a single trifluoromethylation‐induced lactonization (Scheme 18, bottom) [42]. Later, Toda et al. reported improved photocatalyst **PC2** and utilized it in a number of radical‐initiated reactions. Combining photocatalyst **PC2** with Umemoto's reagent **7b**, a handful of trifluoromethylation‐induced lactonizations were achieved (Scheme 18, top) [43]. Although the mechanisms of the reactions depicted on Scheme 18 was not investigated in detail, the radical‐induced nature of other reactions using **PC1/PC2** and illumination was proven; which strongly supports that these reactions follow the general mechanism depicted on Scheme 11 [42, 43].

**SCHEME 19:**
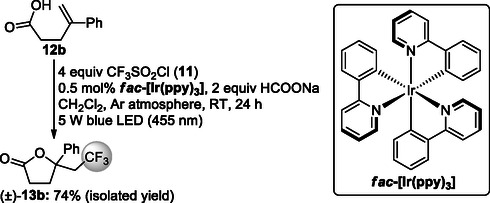
Trifluoromethylation‐induced lactonization of **12b** using triflyl chloride and *fac*‐[Ir^III^(ppy)_3_] photocatalyst.

Han and coworkers reported carboethoxydifluoromethylation‐induced lactonization of terminal alkenoic acids using BrCF_2_COOEt in the presence of *fac*‐[Ir^III^(ppy)_3_] photocatalyst, a base, and irradiation with 455 nm blue LEDs. Addition of radical scavengers like TEMPO or 1,1‐diphenylethylene completely inhibited the reaction (the presence of the CF_2_COOEt‐adducts of the scavengers was confirmed in these reaction mixtures). Based on this and relevant literature data, the authors proposed that the reaction follows a mechanism which is analogous to the one depicted on Scheme 11 (just uses a different fluorinated radical). Encouraged by the success of their protocol, Han and coworkers tried to extend the reaction by replacing BrCF_2_COOEt with precursors of other fluorinated radicals. Amongst these reactions, there was a single trifluoromethylation‐induced lactonization (Scheme 19). Note: in this reaction, HCOONa serves as a base, not as a hydride donor [44].

**SCHEME 20:**
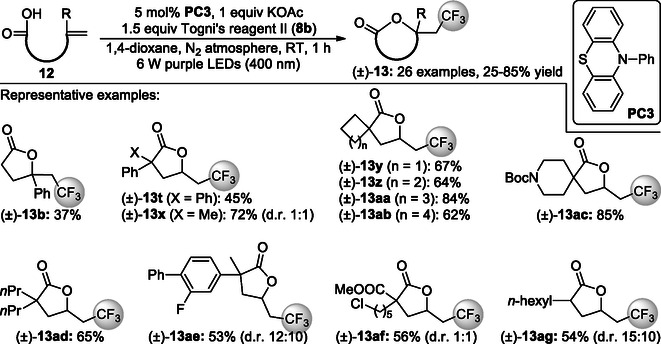
Trifluoromethylation‐induced lactonization of terminal alkenoic acids using Togni's reagent II and *N*‐phenylphenothiazine photocatalyst. Diastereomeric ratios were determined by ^1^H NMR analysis of the crude mixture. Isolated yields are provided.

**SCHEME 21:**
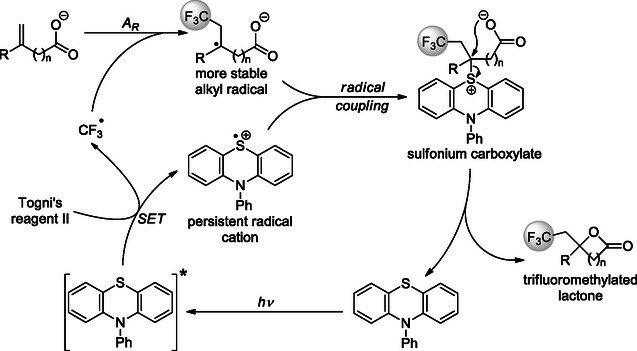
Proposed mechanism of trifluoromethylation‐induced lactonization of terminal alkenoic acids using Togni's reagent II and *N*‐phenylphenothiazine photocatalyst.

Han, Wang, Liao, and coworkers reported trifluoromethylation‐induced cyclization of terminal alkenoic acids (and two unsaturated alcohols) using Togni's reagent II, *N*‐phenylphenothiazine photocatalyst (**PC3**), and illumination with 400 nm purple LEDs (Scheme 20). To elucidate the mechanism, various control experiments were performed. Turning off irradiation halted the reaction, excluding radical chain mechanism. Addition of radical scavenger TEMPO inhibited the reaction (HRMS of such mixtures, however, detected a substrate + CF_3_ + TEMPO adduct). HRMS of a “normal” reaction with substrate **12aa** detected a substrate + CF_3_ + **PC3** adduct. (Note that single‐electron oxidation of *N*‐phenylphenothiazines leads to persistent radical cations). Based on these data, the authors proposed that this reaction initially follows the mechanism depicted on Scheme 11, but halfway through the reaction the trifluoromethylated alkyl radical is not oxidized. Instead, it recombines with the persistent radical cation of the photocatalyst, yielding a zwitterionic sulfonium carboxylate, which alkylates itself to form the product and liberates the photocatalyst (Scheme 21) [45].

As mentioned at the beginning of this section, electrochemical CF_3_ radical‐induced lactonizations are the second biggest group of copper‐free trifluoromethylative lactonizations (out of twelve such methods, four utilize electrochemistry) [46, 47, 48, 49]. The reasons are simple: electrochemical generation of radicals has a controllable rate [46, 55] and does not require excess redox reagents [47, 55], promising a more environmentally friendly and more efficient transformation [55]. Furthermore, redox‐neutral photoredox trifluoromethylation‐induced lactonizations (Scheme 11) can only use trifluoromethylating agents which need single‐electron reduction to provide CF_3_ radicals [38, 39, 40, 41, 42, 43, 44, 45], while electrochemistry enables utilization of Langlois’ reagent (CF_3_SO_2_Na, **10**), which is cheap, easy to handle, and needs single‐electron oxidation to provide CF_3_ radicals. Actually, all four electrochemical methods use CF_3_SO_2_Na and two oxidation substeps (the first generates the CF_3_ radical, while the second transforms the trifluoromethylated alkyl radical intermediate into a trifluoromethylated carbocation, see Scheme 3 for further details) [46, 47, 48, 49]. Note: in order to use CF_3_SO_2_Na as the CF_3_ radical source of a lactonization, the traditional alternative of electrochemistry is utilization of a stoichiometric amount of oxidizing agent, which generates a lot of inorganic waste [36, 37].

**SCHEME 22:**
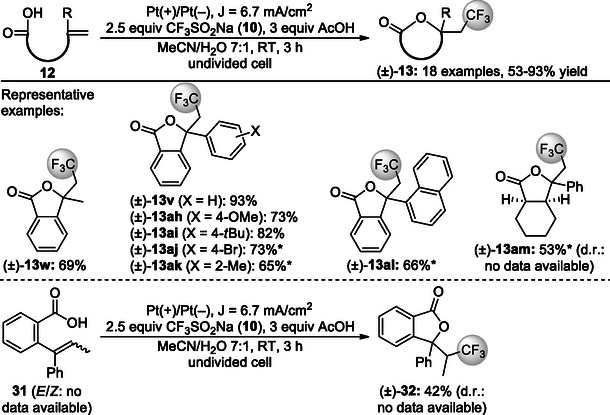
Electrochemical trifluoromethylation‐induced lactonization of terminal alkenoic acids. Isolated yields are provided. Yields marked with asterisk were obtained using CF_3_COOH instead of AcOH.

Zhang, Xu, and coworkers reported electrochemical difluoromethylation‐ and trifluoromethylation‐induced lactonizations of terminal alkenoic acids. The setup is very simple: the substrate, H_(3−*x*)_CF_*x*_SO_2_Na (*x* = 2,3), and some acetic acid additive are dissolved in MeCN/H_2_O mixture, then the mixture is electrolyzed under constant current conditions. From the two processes, the trifluoromethylation‐induced one (Scheme 22) was more efficient. Notably, no data are provided about the diastereomeric ratio of products (±)‐**13am** and (±)‐**32**. Cyclic voltammetry proved that the sulfinates are oxidized more easily than the substrate, and addition of radical scavenger 1,1‐diphenylethylene completely inhibited the reaction, strongly supporting the general mechanism depicted on Scheme 3 [46].

**SCHEME 23:**
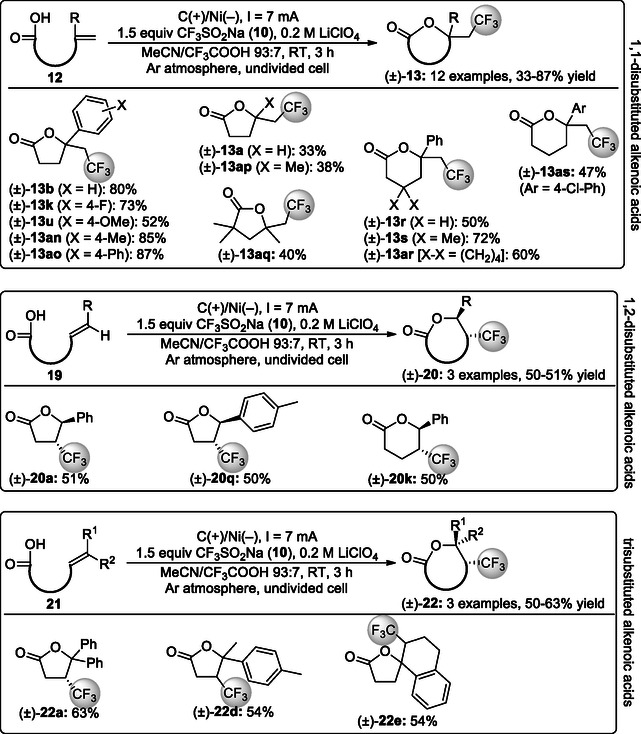
Electrochemical trifluoromethylation‐induced lactonization of terminal (top) and internal (middle and bottom) alkenoic acids. Isolated yields are provided. Synthesis of (±)‐**13u** was performed in MeCN/H_2_O/CF_3_COOH 86/7/7. Transformations of all internal alkenoic acids provided a single diastereoisomer (stereochemistry was not always determined).

Fantoni, Cabri, and coworkers reported electrochemical trifluoromethylation‐induced lactonizations of terminal and internal alkenoic acids. Again, the setup is very simple: the substrate, CF_3_SO_2_Na, and some LiClO_4_ (supporting electrolyte) are dissolved in MeCN/CF_3_COOH mixture, then the mixture is electrolyzed under constant current conditions (Scheme 23). To achieve good yields, the presence of at least one aryl group was necessary on the alkene. Under the same conditions, trifluoromethylation‐induced etherification was also successfully accomplished. Cyclic voltammetry proved that CF_3_SO_2_Na is oxidized more easily than the substrate, and addition of radical scavengers (TEMPO or BHT) completely inhibited the reaction (the BHT‐CF_3_ adduct was detected using HPLC‐MS in such mixtures), strongly implying that the process follows the general mechanism depicted in Scheme 3 [47].

**SCHEME 24:**
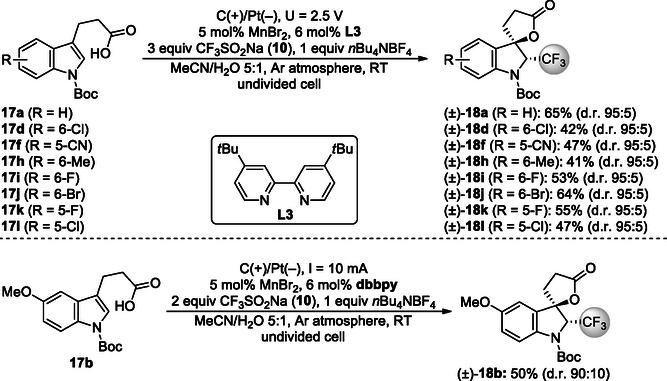
Electrochemical trifluoromethylation‐induced lactonization of 3‐(indol‐3‐yl)propanoic acids. Diastereomeric ratios were determined by ^1^H and ^19^F NMRs of the reaction mixture. Isolated yields of the *trans* product are provided.

Wu and coworkers reported electrochemical synthesis of trifluoromethylated 3,3‐spiroindolines from 3‐(indol‐3‐yl)propane‐1‐ols, *N*‐tosylated 3‐(indol‐3‐yl)propane‐1‐amines, and 3‐(indol‐3‐yl)propanoic acid derivatives. Compared to the electrochemical protocols above, this one used a more complicated setup: apart from the substrate, the CF_3_SO_2_Na, and the supporting electrolyte (*n*Bu_4_NBF_4_), 5 mol% MnBr_2_, and 6 mol% 4,4′‐di‐tert‐butyl‐2,2′‐bipyridyl (**L3**) were also dissolved in the solvent. Some substrates were transformed using constant voltage, while others were transformed using constant current (Scheme 24). The reactions were strongly diastereoselective. Addition of the radical scavenger TEMPO inhibited formation of the trifluoromethylated products, indicating the intermediacy of CF_3_ radicals. To understand the role of the additives, a number of control experiments were performed. Compared with the additive‐free system (substrate, CF_3_SO_2_Na, and *n*Bu_4_NBF_4_ supporting electrolyte in MeCN/H_2_O 5:1), the presence of 5 mol% bromide provided better yields, the presence of 5 mol% MnCl_2_ and 6 mol% **L3** provided much better yields, and the presence of 5 mol% MnBr_2_ and 6 mol% **L3** provided the best yields. This suggests that the reaction follows the general mechanism depicted in Scheme 3, but the oxidation substeps (CF_3_SO_2_Na → CF_3_ radicals; trifluoromethylated alkyl radical → trifluoromethylated carbocation) are mainly performed by electrochemically generated Mn(III) species (not by direct anodic oxidation) [48]. Note: it is experimentally proven that “traditional” chemical systems using excess Mn(OAc)_3_ and CF_3_SO_2_Na can trigger trifluoromethylation‐induced cyclization of indole derivatives with a pendant nucleophilic group [37, 48].

**SCHEME 25:**
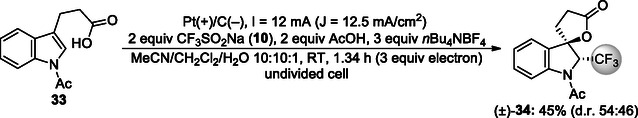
Electrochemical trifluoromethylation‐induced lactonization of 3‐(indol‐3‐yl)propanoic acid derivative **33**. Isolated yield is provided. Diastereomeric ratio was determined by SFC‐MS.

Gao, Zhu, and coworkers reported electrosynthesis of trifluoromethylated spirocyclic indolines. The process was usually highly diastereoselective, but the authors noted that detection of the minor components were often difficult with ^1^H NMR (therefore, they used SFC‐MS instead). The majority of the substrates were 3‐(indol‐3‐yl)propane‐1‐ols. Their trifluoromethylation‐induced etherifications were inhibited by radical scavengers such as TEMPO, BHT, or 1,1‐diphenylethylene, suggesting the involvement of radicals. Cyclic voltammetry indicated that under the reaction conditions, CF_3_SO_2_Na is oxidized more easily than the indole substrates. Furthermore, some substrates underwent C2‐trifluoromethylation instead of trifluoromethylation‐induced etherification. Together, the above data strongly support a mechanism which is analogous to the one depicted on Scheme 3. Amongst the reactions reported by the authors, there was a single trifluoromethylation‐induced lactonization (Scheme 25) [49].

### 3.3 “Electrophilic” Trifluoromethylation‐Induced Lactonizations

**SCHEME 26:**
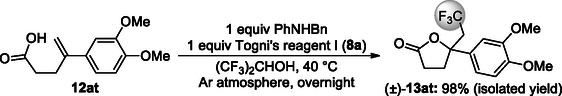
Lactonization of a 4‐arylpent‐4‐enoic acid induced by true CF_3_^+^ transfer.

As mentioned in Section 2, as of now, there is only one known trifluoromethylation‐induced lactonization, which definitely follows the completely ionic pathway depicted in Scheme 2. Colomer and coworkers reported aryltrifluoromethylation of styrenes using anilines and Togni's reagent I (**8a**) in hexafluoroisopropanol as solvent. No product was formed in AcOH, CF_3_COOH, MeCN, or CH_2_Cl_2_ as solvents, while trifluoroethanol provided only a very low amount of product. Replacement of Togni's reagent I (**8a**) with Togni's reagent II (**8b**) halved the yield, while Umemoto's reagent (**7a**) provided no product. The presence of radical scavengers (TEMPO, BHT) did not affect the reaction, ruling out radical pathways. Cyclic voltammetry proved that Togni's reagent I becomes significantly more electrophilic when it is dissolved in hexafluoroisopropanol. DOSY experiments suggest that Togni's reagent I forms stable associates with (CF_3_)_2_CHOH via hydrogen bonding, which could be responsible for activation of the reagent. Based on these data and further kinetic studies, the authors proposed that the reaction starts with CF_3_^+^ transfer from the activated Togni's reagent I to the styrene, and the resulting benzylic carbocation alkylates the aniline. The authors proved that the benzylic carbocation intermediate can capture internal nucleophiles by subjecting styrene derivative **12at** to their standard conditions (Scheme 26): in this reaction, the aniline derivative was only a bystander because intramolecular lactonization was faster than intermolecular aryltrifluoromethylation [50]. It is reasonable to assume that the trifluoromethylation‐induced lactonization depicted on Scheme 26 could proceed in the absence of the aniline, and it may even be extended to other alkenoic acids. However, to date, there has been no further research on the application of Togni's reagent I in (CF_3_)_2_CHOH for trifluoromethylation‐induced lactonizations.

## 4 Summary and Outlook

Although the history of trifluoromethylation‐induced lactonization is less than one and a half decade old, it already developed into a diverse research topic which reflects the creativity and ingenuity of chemists. Below, I provide a concise summary of existing methods and the timeline of their development, which will hopefully illustrate the above point. Afterward, future challenges and opportunities will be discussed.

### 4.1 Concise Summary of Existing Methods

Lactonizations of olefincarboxylic acids are usually electrophile‐induced. However, available electrophilic trifluoromethylating agents are usually unable to transfer CF_3_^+^ to weakly nucleophilic alkenes. (A sole exception was discovered only in 2023) [50]. Thus, for a long time, researchers had to use an alternative pathway: radical‐induced trifluoromethylative lactonization.

The first such method, reported in 2012, utilized Togni's reagent II (an electrophilic CF_3_ source) and an in situ generated Cu(I) complex as the catalyst [31]. Zhu and Buchwald realized that the copper complex may be involved in both CF_3_ radical formation (single electron transfer from Cu(I) to Togni's reagent II provides a Cu(II) ion and a CF_3_ radical) and C—O bond formation (the trifluoromethylated alkyl radical intermediate can be captured by Cu(II), yielding an alkylcopper(III) carboxylate; see Scheme 4 for further details), understood that Cu‐catalyzed C—O bond formation may result in asymmetric lactonization, and successfully developed an enantioselective variant of their protocol as early as 2013 [33].

The next important step was the development of redox‐neutral photocatalyzed CF_3_ radical‐induced lactonization in 2014 [38]. The catalytic cycle was depicted on Scheme 11. Upon excitation, the reduced photocatalyst transfers an electron to the CF_3_ source, resulting in a trifluoromethyl radical and the oxidized photocatalyst. Note: Addition of the CF_3_ radical to the alkene provides the trifluoromethylated alkyl radical intermediate, which then transfers an electron to the oxidized photocatalyst. This regenerates the photocatalyst, and provides a trifluoromethylated carbocation whose ring closure finishes the reaction. Because the reaction conditions are mild, and there is no need for stoichiometric redox agents (this reduces waste formation, which is an important “green” goal nowadays), photoredox catalysis quickly became the most popular way of achieving trifluoromethylative lactonizations [38, 40, 41, 42, 43, 44, 45].

In the meantime, attempts were made to utilize cheap and easy‐to‐handle Langlois’ reagent (CF_3_SO_2_Na) as the CF_3_ radical source of lactonizations. Because generation of CF_3_ radicals from CF_3_SO_2_Na and transformation of the trifluoromethylated alkyl radical intermediate of the lactonization process to a carbocation (see Scheme 3) both require oxidation, utilization of Langlois’ reagent in CF_3_ radical‐induced lactonization needs either a stoichiometric amount of chemical oxidant or anodic oxidation. The first successes were reported in 2017 (two methods which used CF_3_SO_2_Na and a stoichiometric amount of a chemical oxidant) [36, 37].

Later, in 2019, trifluoromethylation‐induced lactonization using anodic oxidation of CF_3_SO_2_Na was accomplished too [46]. Although electrochemistry is a logical choice if generation of radicals is desired, it is an unfamiliar area for most organic chemists, which hinders its applications [55]. Thus, it is understandable that the first electrocatalyzed method for CF_3_ radical‐induced lactonization was reported 5 years after the first photoredox‐catalyzed method (and 7 years after the first successful trifluoromethylation‐induced lactonization) [46]. Yet, electrocatalytic oxidation of CF_3_SO_2_Na is now the second most popular way of generating CF_3_ radicals for lactonizations [46, 47, 48, 49], because it can generate radicals at a controllable rate and does not require excess redox reagents (reducing waste formation).

Finally, in 2023, the first (and to date, the only) CF_3_^+^ transfer‐induced lactonization was reported [50]. It follows a completely ionic mechanism, without any involvement of radicals.

### 4.2 Future Challenges and Opportunities

Overall, trifluoromethylation‐induced lactonization enables fast and efficient synthesis of trifluoromethylated lactones, opening a convenient pathway for access to new fluorinated building blocks. Taking into account the interest in new methods for synthesis of trifluoromethylated compounds (fueled by the increasing importance of trifluoromethylated drugs) [2, 3, 17], further development of this area is expected. Based on current trends in synthetic chemistry, the most likely goals will be extension of the substrate scope (many current methods provide reduced yields if the intermediate trifluoromethylated radical/carbocation is not benzylic), development of new asymmetric methods, utilization of milder reaction conditions, improvement of functional group tolerance, reduction of environmental impact (greener chemistry), and utilization of cheaper CF_3_ sources.

Concerning CF_3_ sources, traditional electrophilic trifluoromethylating agents (Umemoto's reagents and Togni's reagents) are quite expensive, while radical trifluoromethylating agents (CF_3_Br, CF_3_I, CF_3_SO_2_Na, CF_3_SO_2_Cl) are much more affordable [54]. The price of a recently commercialized electrophilic trifluoromethylating agent, *S*‐(trifluoromethyl)thianthrenium triflate [56], falls somewhere between the above two groups. Because almost all trifluoromethylative lactonizations are CF_3_ radical‐induced, their ideal CF_3_ source should not readily participate in reactions other than CF_3_ radical generation, should be stable, should be cheap, and should be easy to handle. In my opinion, Langlois’ reagent (CF_3_SO_2_Na) and *S*‐(trifluoromethyl)thianthrenium triflate fulfill all these criteria, CF_3_Br and CF_3_I fulfill most criteria except handling (their gaseous nature complicates their handling), while triflyl chloride partially fulfills most criteria except reactivity (it is a strong sulfonylating agent, which can cause side reactions) and, to an extent, handing (because of its volatile nature, this liquid is a bit more difficult to handle than solids). It is important to note, however, that Langlois’ reagent provides CF_3_ radicals upon oxidation, while CF_3_Br, CF_3_I, triflyl chloride, and *S*‐(trifluoromethyl)thianthrenium triflate provide CF_3_ radicals upon reduction. Thus, the choice of the reagent depends on the exact reaction mechanism too.

Now, let's discuss possible future developments of various methods. Trifluoromethylation‐induced lactonization using Togni's reagent II and a Cu(I) catalyst provides good yields, does not require extra redox agents [31, 32, 33], and can be performed with good enantioselectivity in the presence of an appropriate chiral ligand [33]. The latter is especially important, because no other protocol has yet been able to achieve asymmetric trifluoromethylative lactonization. Therefore, the greatest improvement would be replacing expensive Togni's reagent II with cheaper alternatives. Current Cu‐catalyzed protocols generate CF_3_ radicals by reduction, so, in theory, *S*‐(trifluoromethyl)thianthrenium triflate, CF_3_Br, CF_3_I, and triflyl chloride could be viable CF_3_ sources. Note: these reagents have different redox potential than Togni's reagent II, so they may require a different Cu(I) complex as catalyst.

Redox‐neutral photocatalyzed trifluoromethylative lactonizations were realized with a variety of photocatalysts [38, 40, 41, 42, 43, 44, 45]. They have a number of advantageous properties (yields are usually good, reaction conditions are mild, waste formation is reduced), but the majority of these reactions generate CF_3_ radicals by reduction of expensive reagents (Umemoto's reagent [38, 40, 42, 43] or Togni's reagent II [45]). Thus, it would be desirable to develop methods which utilize cheaper CF_3_ sources (CF_3_Br, CF_3_I, *S*‐(trifluoromethyl)thianthrenium triflate, or CF_3_SO_2_Cl). Notably, there is already an efficient method which utilizes CF_3_Br [41], and there are two reactions [40, 44] which proved that triflyl chloride is a viable CF_3_ source in redox‐neutral photocatalyzed trifluoromethylative lactonizations. More such methods are expected in the future. Another intriguing (although not necessary simple) possibility is incorporation of Cu(II)‐catalyzed C—O bond formation into these methods to develop enantioselective protocols. In the mechanisms of known photoredox lactonizations, C—O bond formation is unassisted; therefore, only racemic products can be formed. It is proven that in the presence of a chiral ligand, Cu(II)‐catalyzed C—O bond formation can be enantioselective [33].

Methods which use cheap CF_3_SO_2_Na and a stoichiometric amount of chemical oxidant to achieve CF_3_ radical‐induced lactonization [36, 37] may be more convenient to many organic chemists than electrocatalytic methods, but they generate lots of waste. Utilization of more environmentally friendly oxidants may alleviate this problem. It may also be worth testing these protocols in the presence of chiral Cu(II) complexes. Chiral Cu(II) species could react with the trifluoromethylated alkyl radical intermediate to form a chiral alkylcopper(III) carboxylate, whose reductive elimination would provide the lactone product in an enantioenriched form as well as a chiral Cu(I) species. Then, the chemical oxidant could oxidize the Cu(I) species back to a Cu(II) species, and the catalytic cycle can start again.

Methods which use anodic oxidation of CF_3_SO_2_Na to achieve trifluoromethylative lactonization [46, 47, 48, 49] are very promising: even quite simple setups provide good yields [46, 47], the utilized CF_3_ source is inexpensive and easy to handle, the key radicals can be generated in a controllable rate, and waste formation is greatly reduced (especially compared to methods that use the chemical oxidation of CF_3_SO_2_Na). Thus, further electrocatalytic methods are expected in the future. Unfortunately, current electrocatalytic trifluoromethylative lactonizations are only suitable for the synthesis of racemic products. There is a chance, however, that addition of an appropriate chiral copper complex to the reaction mixture may result in enantioselective trifluoromethylative lactonization (for more details, see the paragraph above).

There is one unique trifluoromethylation‐induced lactonization reaction which follows a completely ionic mechanism, without any involvement of CF_3_ radicals [50]. It may be the first member of a novel group of trifluoromethylative lactonization protocols. However, because it requires quite specific (and costly) conditions to provide a good yield (expensive Togni's reagent I in expensive hexafluoroisopropanol as solvent), there is also a reasonable chance that it will remain merely a curiosity. Only time will tell.

## Funding

This study was supported by Hungarian Scientific Research Fund (Grant NKFI K138871), the Ministry of Innovation and Technology of Hungary (Grant TKP2021‐EGA‐32), and the University of Szeged Open Access Fund (Grant No. 8930).

## Conflicts of Interest

The author declares no conflicts of interest.

## Data Availability

Data sharing not applicable to this article as no datasets were generated or analyzed during the current study.
